# A central role for amyloid fibrin microclots in long COVID/PASC: origins and therapeutic implications

**DOI:** 10.1042/BCJ20220016

**Published:** 2022-02-23

**Authors:** Douglas B. Kell, Gert Jacobus Laubscher, Etheresia Pretorius

**Affiliations:** 1 Department of Biochemistry and Systems Biology, Institute of Systems, Molecular and Integrative Biology, Faculty of Health and Life Sciences, University of Liverpool, Liverpool L69 7ZB, U.K.; 2 The Novo Nordisk Foundation Centre for Biosustainability, Technical University of Denmark, Kemitorvet 200, 2800 Kgs Lyngby, Denmark; 3 Department of Physiological Sciences, Faculty of Science, Stellenbosch University, Stellenbosch Private Bag X1 Matieland, 7602, South Africa; 4 Mediclinic Stellenbosch, Stellenbosch 7600, South Africa

**Keywords:** amyloid, clotting, COVID

## Abstract

Post-acute sequelae of COVID (PASC), usually referred to as ‘Long COVID’ (a phenotype of COVID-19), is a relatively frequent consequence of SARS-CoV-2 infection, in which symptoms such as breathlessness, fatigue, ‘brain fog’, tissue damage, inflammation, and coagulopathies (dysfunctions of the blood coagulation system) persist long after the initial infection. It bears similarities to other post-viral syndromes, and to myalgic encephalomyelitis/chronic fatigue syndrome (ME/CFS). Many regulatory health bodies still do not recognize this syndrome as a separate disease entity, and refer to it under the broad terminology of ‘COVID’, although its demographics are quite different from those of acute COVID-19. A few years ago, we discovered that fibrinogen in blood can clot into an anomalous ‘amyloid’ form of fibrin that (like other β-rich amyloids and prions) is relatively resistant to proteolysis (fibrinolysis). The result, as is strongly manifested in platelet-poor plasma (PPP) of individuals with Long COVID, is extensive fibrin amyloid microclots that can persist, can entrap other proteins, and that may lead to the production of various autoantibodies. These microclots are more-or-less easily measured in PPP with the stain thioflavin T and a simple fluorescence microscope. Although the symptoms of Long COVID are multifarious, we here argue that the ability of these fibrin amyloid microclots (fibrinaloids) to block up capillaries, and thus to limit the passage of red blood cells and hence O_2_ exchange, can actually underpin the majority of these symptoms. Consistent with this, in a preliminary report, it has been shown that suitable and closely monitored ‘triple’ anticoagulant therapy that leads to the removal of the microclots also removes the other symptoms. Fibrin amyloid microclots represent a novel and potentially important target for both the understanding and treatment of Long COVID and related disorders.

## Introduction

In many cases, individuals infected with the SARS-CoV-2 virus and suffering from COVID-19 continue (or in some case begin) to display symptoms long after the acute phase. Depending on the demographics of the hosts and variant of SARS-CoV-2, these post-acute sequelae of COVID-19 (PASC [1]) may affect ∼30% of all infected individuals [2,3], are starting to be observed even in children [4,5], and are commonly known as long COVID [6–10]. Many regulatory health bodies still do not recognize this syndrome as a separate disease entity, and refers to it under the broad terminology of ‘COVID’. The symptoms of long COVID are multifarious [11,12] and include breathlessness, fatigue, chest pain, myalgia, cognitive dysfunction, innate immune responses coupled to inflammatory cytokine production, and a pro-coagulant state. Our focus is on the latter.

At present there is no established treatment for long COVID (e.g. [13,14], so from a systems point of view it is important to understand which symptoms are ‘primary’, and which are simply secondary effects of the primary symptoms themselves. This would allow treatment strategies to focus on the primary symptoms and their causes. We here make the case (with evidence) that much of the aetiology of long COVID can be attributed to the formation of aberrant amyloid fibrin microclots, triggered in particular by the SARS-Cov-2 spike protein, and that by inhibiting the transport of erythrocytes to capillaries, and hence O_2_ transfer, it is these amyloid microclots that are mainly responsible for the various long COVID symptoms observed. The microclots may also present novel antigens that lead to the production of autoantibodies, that can exacerbate symptoms further. This understanding of the role of such microclots may be expected to lead to an effective strategy for treating long COVID (and probably for other, related conditions such as myalgic encephalomyelitis/chronic fatigue syndrome (ME/CFS)).

## Protein conformation, prions, and amyloid structures

**Figure 1.**
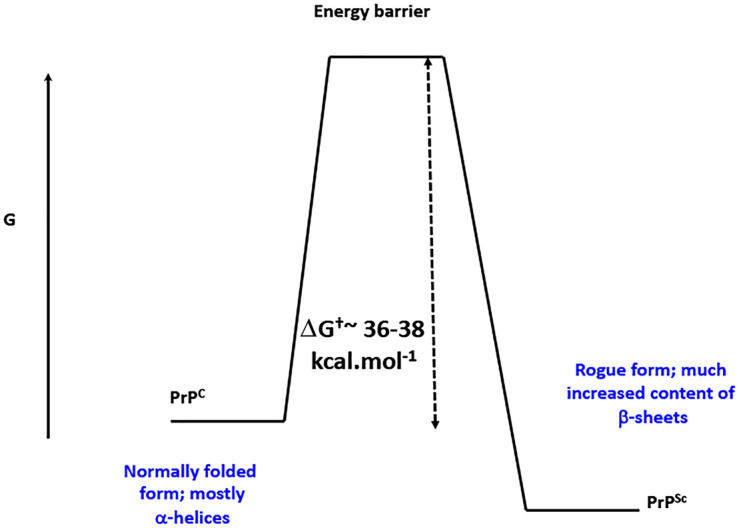
Many proteins can adopt more than more thermodynamically stable microstate with no change in primary structure (sequence), in which the more stable contains an ordered β-sheet ‘amyloid’ structure. Normally, however, it is present in a less stable state that is kinetically more accessible during and following its synthesis. The more stable (labeled PrP^Sc^) is separated from the initial state (PrP^C^) via a large energy barrier. This is true for amyloid proteins generally, and is illustrated here for classical prion proteins. Redrawn from a CC-BY publication at [26].

Classically, it was assumed that proteins folded into their conformational states of lowest free energy, because unfolded proteins typically refolded into their original forms [15]. However, the discovery of prion proteins in particular showed that this was not always the case; the more common and native form of the prion protein PrP^C^ could be converted — with no change in primary sequence — into a thermodynamically more stable, ‘rogue’ version referred to as PrP^Sc^ (Figure 1), and this transformation was itself catalyszd by PrP^Sc^ (e.g. [16,17]). These ‘rogue’ versions commonly contain a large number of ordered β-sheets in a cross-β architecture [17–23], and are referred to generally as amyloid forms. A great many proteins are now known to be able to adopt such amyloid forms, and over 50 have been implicated in a variety of diseases (‘amyloidoses’) [24–26]. These kinds of amyloid structures may be stained with fluorogenic dyes [27,28] such as congo red [29], thioflavin T [30–35], which is much more visible than congo red, or a variety of conjugated oligothiophenes [36] commercialized as Amytracker^TM^ dyes (e.g. [37,38]).

## Amyloidogenic blood clotting

**Figure 2.**
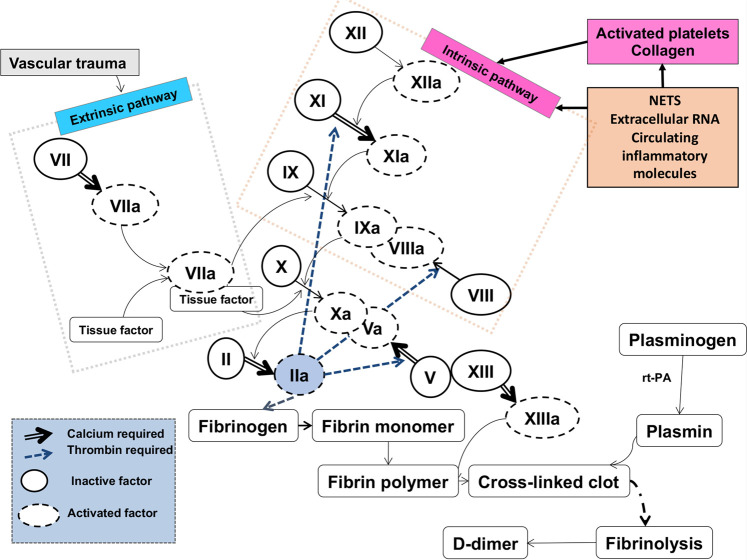
Representation of the classical blood-clotting cascades, ending in the removal of two fibrinopeptides from fibrinogen, and its self organization to produce fibrin (that may then be cross-linked). The eventual result of fibrinolysis, that acts as a record of its extent, is d-dimer. Redrawn from a CC-BY publication at [26].

The normal blood clotting cascade is well established (e.g. [39–41] (Figure 2), with the terminal stages of both ‘intrinsic’ and ‘extrinsic’ pathways involving the self-organised polymerization of soluble fibrinogen to insoluble fibrin, catalysed by thrombin [42]. Fibrinogen, a cigar-shaped molecule of some 5 × 45 nm, is one of the most abundant proteins in plasma (commonly present at 1.5–3.5 g L^−1^ [41]).

**Figure 3.**
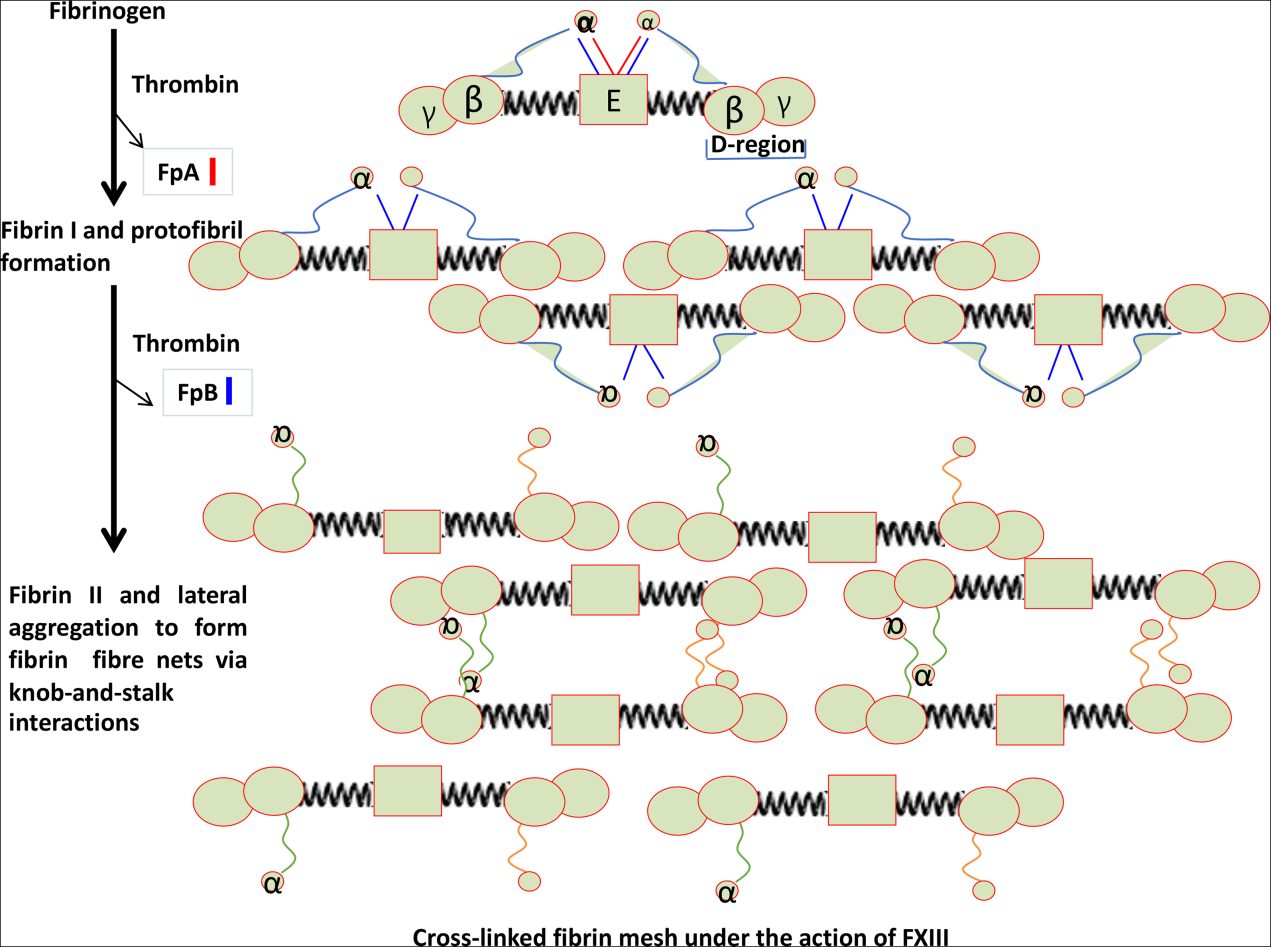
Self organization of fibrinogen into fibrin under the action of thrombin in removing two fibrinopeptides. Redrawn from a CC-BY publication at [26].

The action of thrombin on fibrinogen leads to the removal of two small fibrinopeptides (Figure 3), and this initiates the thermodynamically favorable conversion to fibrin, first as small protofibrils and then as long fibrils, typically with a diameter of 50–100 nm. Factor XIII is a transglutaminase which inserts cross-links into the developing clot, and also serves to cross-link α2-antiplasmin (α2-AP) to fibrin. α2-AP and other similar molecules are major inhibitors of plasmin, the enzyme which is mainly responsible for clot proteolysis (fibrinolysis).

The structure of fibrin clots is typically characterized by the diameter of the spaghetti-like fibres and by the size of the residual pores [43]. However, in some cases these spaghetti-like structures with pores are absent. We initially characterized this phenomenon, observed in the electron microscope, as ‘dense matted deposits’ (e.g. [44–46]). We later discovered that such structures could be induced by highly substoichiometric amounts of bacterial lipopolysaccharide (LPS) (1 molecule LPS per 100,000,000 fibrinogen molecules), and it emerged that they too were, in fact, amyloid in character [26,47–50]. This highly substoichiometric induction (of a thermodynamically favourable process) is very important, as it effectively provides a means for a massive amplification of what may be a tiny amount of trigger material.

The list of molecules that could induce this anomalous clotting, additional to the bacterial LPS that was then our main focus, included iron ions [51–56], oestrogens [46,57], lipoteichoic acid [49,52], and serum amyloid A [58].

While the list of the many other molecules that can plausibly effect this anomalous amyloid-type clotting is unclear, such clots may also be observed in the blood of individuals with inflammatory diseases such as Alzheimer's [37,50,59–61], Parkinson's [37,48], type 2 diabetes [37,38,62–64], and rheumatoid arthritis [65–68]. Similar phenomena have also been observed in the pregnancy disorder pre-eclampsia [69], where there is also strong evidence for a microbial component [70,71].

Although this was a novel finding for ‘normal’ blood, it had previously been shown that β-sheet structures could be induced in fibrin artificially by mechanical means [72]. There have also been rare reports (see [26,73,74]) of amyloidogenic mutations (alleles) in the fibrinogen Aα chain. Additionally, it has long been known that aged fibrin deposits can bind amyloid dyes in tissue sections [75] and that fibrin-derived peptides can form cross-beta structures [76]. The amyloid form of the prion protein is highly resistant to proteolysis (resistance to proteolysis by proteinase K is used in an assay for PrP^Sc^ [77–79]), and so this provided a ready explanation for the different nature and persistence of the amyloid fibrin clots [52].

### A note on ‘amyloid’ terminology

We recognize that there has occasionally been confusion in our use of the term ‘amyloid’ to describe amyloidogenic blood clotting, as blood is not seen as a source of the classical amyloidoses. While we could have used another term, the structures and staining of these blood clot fibres, presumably consisting of the ordered β-sheet architectures necessary (i) to bind fluorogenic amyloid stains and (ii) to be resistant to the normal sources of proteolysis, do reflect the well-established term ‘amyloid’. In addition, there is a very simple discrimination between ‘classical’ amyloids and those fibrin-rich amyloids that are our focus here: this is that the amyloid fibrils seen in classical amyloidoses tend to be ca 5–25 nm or so in diameter [21,22,80,81] whereas those in fibrin amyloid microclots tend to be in the range 50–150 nm or even more. They are thus easily distinguished microscopically, even without the greater analytic power afforded by proteomics [82], antibody staining [83], and so on. However, to avoid such ambiguity in the future, we consider it sensible to refer to the kinds of fibrin-based amyloids we are speaking about as fibrinaloids.

## Amyloid fibrin microclots (fibrinaloids) in COVID-19 and long COVID

**Figure 4.**
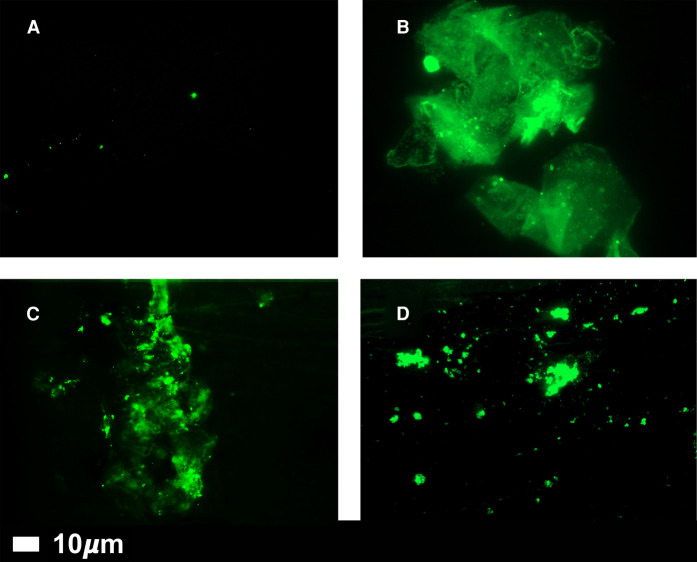
Fluorescence microscopy of representative micrographs showing microclots in the circulation of controls (A) and in patients with Long COVID (B–D). Absence of significant amyloid microclots in the plasma of ‘normal’ individuals, and their significant presence in the plasma of individuals with long COVID. Platelet-poor plasma was produced by centrifugation at 3000×***g*** for 15 min, stained with 5 µM thioflavin T, and imaged in a fluorescence microscope (Zeiss Axio Observer 7 with a Plan-Apochromat 63×/1.4 Oil DIC M27 objective (Carl Zeiss Microscopy, Munich, Germany). Wavelengths were Exc 450–488 nm/emission 499–529 nm, all as in [108].

Coagulopathies [84–102], and especially the formation of extensive microclots *in vivo*, are a hallmark of both COVID [85,103–115] and long COVID [116,117], and we have demonstrated that these microclots too are amyloid in character [108,109,116]. Importantly, the addition of purified, recombinant SARS-CoV-2 S1 spike protein to coagulation-competent normal plasma is sufficient to induce the formation of anomalous clots [118] that adopt amyloid states that are also resistant to fibrinolysis [108]. Note that the observations of the microclots in (platelet-poor) plasma are performed without the addition of exogenous thrombin; they are naturally there in the circulation of patients with both acute and long COVID. The size of these amyloid microclots, that can be observed microscopically and stained e.g. with thioflavin T [108,109,116] (Figure 4; control vs LC plasma), are typically anywhere from 1–200 µm; this means that they can effectively block up, and inhibit blood flow through, all kinds of microcapillaries, thereby strongly lowering the availability of oxygen in tissues. As expected, they consist mainly of fibrin, but also contain many other proteins, including α2-antiplasmin [108] (and even the virus itself [119]). They also have heightened pro-inflammatory activity and elicit fibrin autoantibodies [118] (and maybe others). Elements of at least some spike protein variants of SARS-CoV-2 can also stimulate (*in silico*) the amyloidogenic aggregation of serum amyloid A [120]. More generally, there is considerable evidence for the induction of amyloid production by amyloidogenic proteins by viruses such as those of the Herpesviridae family, e.g. in Alzheimer's disease [121–125].

###  ‘Ground-glass’ opacities in COVID and long COVID

One of the early hallmarks of acute COVID-19 was the observation of extensive, diffuse opacities in high-resolution computed tomography (HRCT) scans of the lungs of patients, consistent with the breathing difficulties widely observed. These opacities resembled ground glass [126], and indeed are commonly referred to as ‘ground-glass opacities’ (GGO) [127–132]. They are largely formed of fibrin, though we are not aware that anyone has yet stained them for amyloid structures (which is what we believe they must be, and there is ample precedent for this in other diseases (e.g. [133–136])). In COVID patients they persist well beyond the acute phase [137].

### More on the size and properties of microclots

As mentioned, the fibrinaloid microclots that we observe are typically in the range 1–200 µm on their longest axis. This is consistent with the ‘ground glass’ appearance, It is also true for ‘artificial’ amyloid-type protein structures [28], and for the kinds of amyloid seen as deposits in thrombotic microangiopathies [138–142]. However, the size distribution differs markedly between individuals (Figure 4), and while hard to pin down it does provide a ready general explanation for the very different manifestations of Long COVID (including suggestions that the term covers ‘multiple’ diseases). This is also true for prion diseases, where specific ‘strains’ based on particular conformations can propagate in the same form. In some cases, there can be differential staining of individual amyloids by different dyes [26,49,143–145], which also allows a certain degree of differentiation of the structure of the clots. In the case of Long COVID, we are probably not quite ready for such subtleties.

## Role of d-dimer

**Figure 5.**
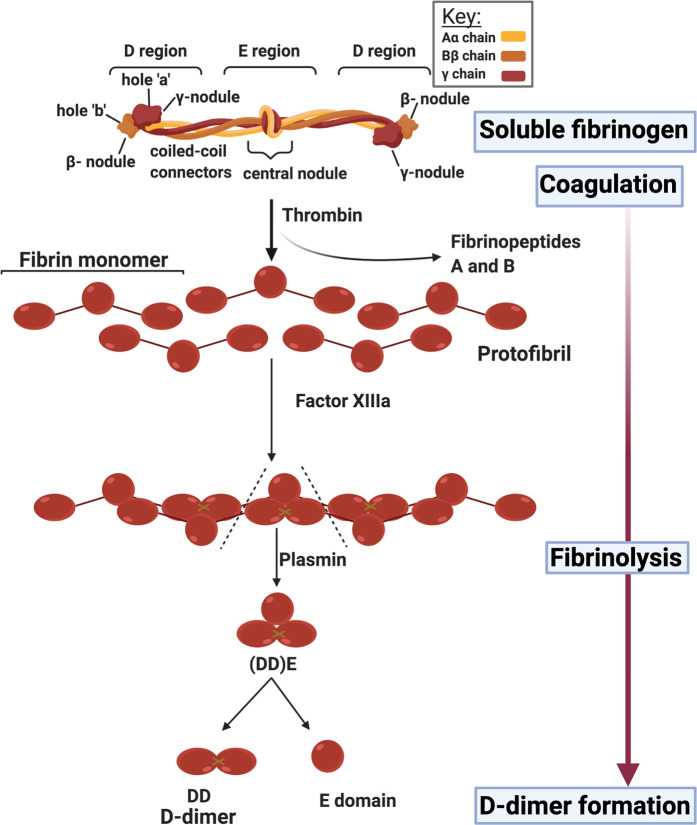
d-dimer production by fibrinolysis. It is believed to be similar when produced from fibrinaloid clots, but the rate is considered to be slower. Image created with BioRender (https://biorender.com/).

**Figure 6.**
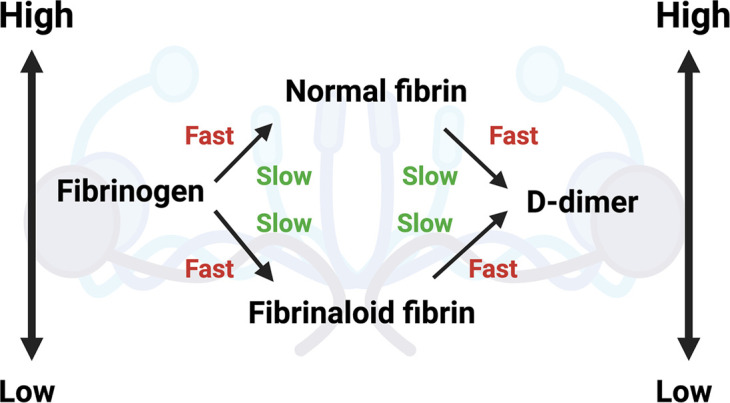
d-dimer levels reflect both the rate of production and rate of degradation of clots, whether the clots are ‘normal’ or fibrinaloid in nature. Image created with BioRender (https://biorender.com/.

**Figure 7.**
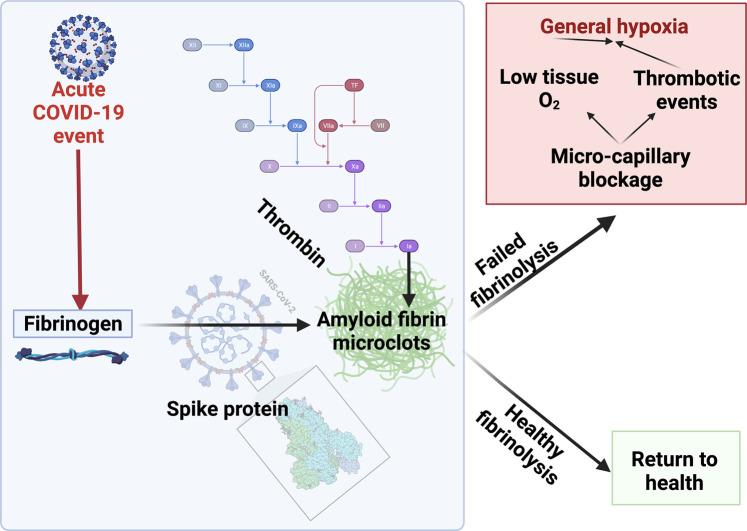
A simplified diagram to explain microclot formation that might either be resolved via fibrinolytic processes after acute COVID-19 or, in some patients, result in a failed fibrinolytic process. Image created with BioRender (https://biorender.com/).

A major marker for fibrinolytic activity (Figure 2) is a polypeptide referred to as d-dimer Figure 5. It is a strong prognostic indicator for disease outcome (survival) in acute COVID [85,146–154]. It is, in effect, a composite measure of how much fibrinogen there was, how much was converted to fibrin clots (whether normal or fibrinaloids), and how much these then went on to be lysed. Each of these general steps may of course be regulated independently (i.e. proceed at a ‘fast’ or slow’ rate, as encoded simplistically in Figure 6), so the analysis of d-dimer measurements must take all of these issues into account. Thus high levels of d-dimer must of necessity represent the production of many more clots (and sufficient fibrinolysis to be occurring) but cannot of themselves reflect how many remained after their lysis nor which of them were ‘normal’ and which amyloid (see Figure 7 for a simplified pathway explanation).This explains why d-dimer levels are significantly raised following infection with many of the earlier variants of SARS-CoV-2, which produce multiple fibrinaloid microclots and Long COVID. However, d-dimer is massively increased in the case of the omicron variant where much clotting takes place but seemingly not to a fibrinaloid form, and thus effective clot fibrinolysis takes place. That there may be strong dependencies on the precise variant is not really surprising (omicron differs from both alpha and delta variants by more than 20 mutations [155], more than enough to vary a protein's activity 1000-fold in typical directed evolution experiments [156]). High d-dimer thus may, but does not have to [157], reflect disease severity given a particular SARS-CoV-2 variant; it depends on the context, and in particular the type of fibrin that can serve as the substrate for its production.

## Assessment of clotting

### Why TEG rather than APTT, INR and other clotting measures?

A variety of optical clotting tests are in more-or-less common usage [158]. These include the aPTT (activated partial thromboplastin clotting time), prothrombin time (PT) and International normalized ratio (INR), but they are considered to have limited predictive value for bleeding and are not cost-effective [159,160]. INR and APTT are of limited value to predict clotting (and identifying patients that will bleed) in patients with acute and Long COVID. The test uses platelet poor plasma after discarding the cellular component of blood (including the platelets).

**Figure 8.**
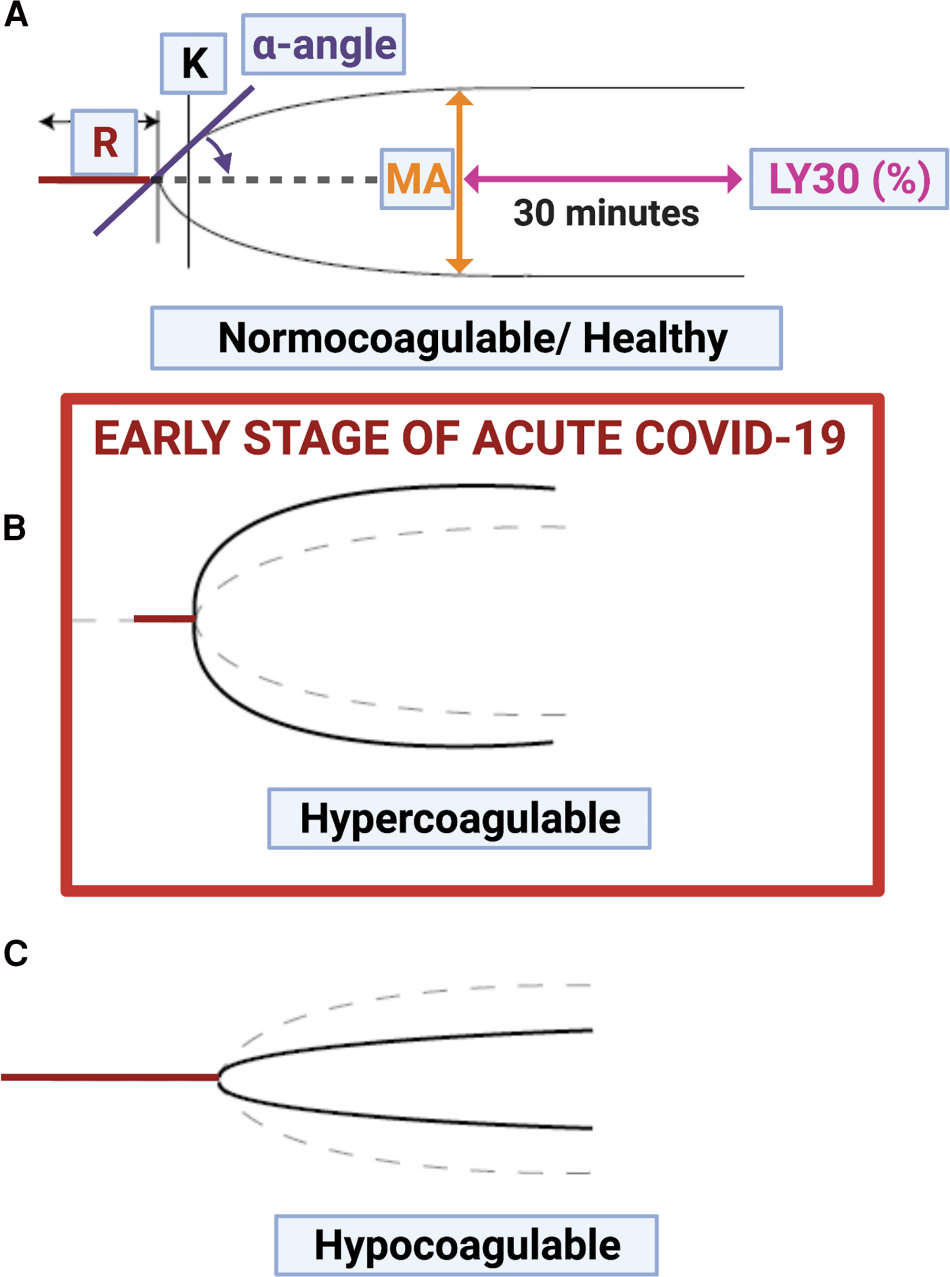
TEG® traces with the main parameters visualized. (**A**) Healthy (normocoagulable) trace; (**B**) Hypercoagulable trace (seen during early stages of acute COVID-19) and (**C**) Hypocoagulable trace. Image created with BioRender (https://biorender.com/). In **B** and **C** the dotted lines represent the normocoagulable case. R represents the time taken to initiate clot formation, α and K reflect the rate and extent of clot formation (e.g. [52]).

As blood clots, the viscosity of the clot increases, and measuring the time-dependent rate and extent of this viscoelasticity change directly in whole blood provides a more comprehensive and convenient *in vitro* assessment of the clotting behavior of the blood at the time. Note that clots of fibrinaloid that have already formed are effectively inert and invisible to such methods. Here the method of choice is known as Thromboelastography (TEG) [48,52,56,58,59,65,66,108,109,161–165] (or a related version known as ROTEM [166–173]). Figure 8 shows stylized traces of TEG, as typically found in health and disease. There are three main generalized traces: healthy (normocoagulable), hypercoagulable (as commonly seen in acute COVID-19) and hypocoagulable (during bleeding risk).

The TEG is a well-known point-of-care method, lately suggested as a good method for following blood clotting status in acute COVID-19, especially when anticoagulation regimes are prescribed to patients [109]. However, in our experience, it is not useful for determining (hyper)coagulation status in Long COVID, and should be reserved for those Long COVID patients where there is either a bleeding concern or actual bleeding. TEG should then be used in conjunction with platelet and microclot mapping, to follow patients closely when bleeding might be expected. Because microclots already contain clotted plasma proteins, and TEG only measures unclotted (soluble) plasma proteins, the TEG might not be suitable to determine the actual existing coagulation status (consisting of both clotted and soluble plasma proteins).

TEG trace ranges might in fact be within normal ranges, and our take on this is that the fibrin inside the microclots has already been coagulated, and trapped inside the microclots, leaving only a lower amount of soluble fibrinogen that might partake in the TEG assay. Therefore both the already-coagulated fibrin in the microclots and the soluble fibrinogen levels should in fact be considered, and preferably measured. Unfortunately the TEG cannot do that*.* However, we consider that TEG is vital for assessing the potential for coagulation when Long COVID patients are treated with anticoagulation therapy, or in any individual undergoing treatment designed to lower the levels of fibrinaloid microclots. In our view, TEG is therefore a much more true evaluation of the patient's coagulation state, as it uses whole blood, thus bringing in the very important role played by platelets.

INR, APTT d-dimer, fibrinogen levels and platelet count may be of help in the very late event of end stage acute COVID-19, where acute disseminated intravascular coagulopathy (DIC)-coagulopathy is seen. Interestingly, it is now accepted that DIC-bleeding events are uncommon in acute COVID-19 [111]. Even in this event, TEG is still of more use to manage this situation [109].

## Mechanical properties of amyloid microclots

While we do not yet know the details of the mechanical properties of the fibrin amyloid microclots, it is known that amyloid fibrils can typically exhibit unusually high mechanical strength and resistance to deformation (e.g. [174–177]. The stiffness also increases with the thickness of the fibres [178]. This implies strongly that the fibrinaloids are likely to be more prone to ‘getting stuck’ in capillaries.

## Bradford Hill criteria and microclots

The Bradford Hill criteria for causation of a disease Y by an environmental factor X [70,179] represent a useful framework for assessing the role of fibrin amyloid microclots in Long COVID, and are as follows: (1) strength of association between X and Y, (2) consistency of association between X and Y, (3) specificity of association between X and Y, (4) experiments verify the relationship between X and Y, (5) modification of X alters the occurrence of Y, and (6) biologically plausible cause and effect relationship. We think that the evidence is very strongly consistent with these criteria when X is represented by microclots and Y by Long COVID and potentially a variety of other conditions.

## Ability of amyloid microclots to explain the symptoms of long COVID

While long COVID is a multi-system disorder with multiple symptoms of varying severity, it remains possible that there is in fact a particular major underlying cause (or that a very small number are the main contributors). Our view is as follows: given that amyloid microclots can clog up capillaries and inhibit the transport of O_2_ to tissues, this alone can in fact more or less self-evidently serve to explain a great many observations, and in particular the symptoms of both acute and long COVID. These obviously include breathlessness due to low O_2_ directly, and thrombotic events such as acute myocardial infarction [180,181], stroke [181–183], etc due to the microclots. The lack of O_2_ transport to tissues explains straightforwardly how a nominally respiratory disease also leads to the dysfunction of organs such as the kidney [184,185], PoTS (postural tachycardia syndrome [186]), myalgia in skeletal muscle [187,188], neurological disorders [189], and lactic acidosis [190,191] (the mass of lactate is too low to have been detected in our own COVID untargeted metabolomics experiments [192]), and potentially the benefits of hyperbaric O_2_ therapy [193]. It is also worth stressing that amyloid structures themselves tend to be cytotoxic, often via membrane disruption [194–198].

### What makes long COVID long?

For any deterministic system to change its behavior there has to be a change of its parameters [199]. In the case of long COVID we need mechanisms that can explain how something that was initiated a long time ago can somehow persist. One source of the continuous production of a stimulus is represented by microbes, including virions, that persist in a largely dormant state (often in intracellular reservoirs) but can occasionally continue to replicate [1,200]. There is now considerable evidence for the persistence of SARS-CoV-2 [201]. Another is the continued release of sequestered microbially derived substances than can act as stimuli for continuing microclot formation. Here, the finding [202] that S1 spike protein can itself persist in CD16^+^ Monocytes in PASC for up to 15 months post-infection is highly relevant, as the amplification of trigger proteins to make microclots as part of the clotting mechanism means that miniscule (and highly substoichiometric) amounts of suitable triggers can suffice [26,47]. This alone is sufficient to account for the chronic nature of such diseases.

### Microclot sequestration of biomarkers

An extra consequence of the production of microclots that sequester other proteins whose concentration would otherwise appear elevated is that those proteins do not then appear in plasma from which the microclots have been removed (e.g. by centrifugation), and thus they do not manifest, and cannot usefully be used, as biomarkers. Thus, our experience [108,116] is (i) that many such proteins including α2-AP and autoantibodies are so sequestered, and (ii) that the degradation of these protease-resistant microclots for classical proteomic analyses requires multiple rounds of trypsinization.

### The role of autoantibodies and biomimicry

Autoantibodies are a feature of many chronic, inflammatory diseases, such as rheumatoid arthritis [68] (where *Proteus* spp are strongly implicated and where the cross-reactive epitopes leading to ‘mimicry’ by and of host protein targets are understood [68,203,204]). Importantly, it has already been shown that both acute [205,206] and Long COVID [207] are accompanied by immunological dysfunction, and by novel antibodies [1,206], including in the latter case to (an ‘abnormal’ but unspecified form of) fibrin [118]. We recognise that any change in the conformation of a protein can result in the generation of novel epitopes that can thereby lead to the production of novel antibodies; indeed the use of secondary antibodies in the detection of small molecules [208] relies precisely on this fact. Although we consider that the more primary event is the generation of fibrinaloid microclots, we also recognise that they are likely to be able to change the natural conformation of many proteins that might normally present as harmless (seen as ‘self’). However, the detailed nature of these autoantibodies is not known (but a subject of considerable present interest [1]).

### Epidemiological aspects

One approach to understanding the mechanisms of long COVID is to analyse epidemiological data, since the characteristics of those experiencing acute COVID differ markedly from those with long COVID [11]. Among these (and in significant contrast with the case of acute COVID [209]), is a striking over-representation of long COVID in women, and especially younger women [3,10,210]. From this point of view, it is of considerable interest that female sex hormones can induce anomalous blood clotting [46,57,211].

Haemophiliacs (and others with hypocoagulatory disorders) represent a complex test case, because depending on the stage of the disease [85], COVID can be both hyper- and hypocoagulatory [212]. However, we are not presently aware of any analyses of fibrinaloid microclots in such individuals.

### Strain differences

While it has been somewhat slowed by the generally sluggish recognition of Long COVID, it is to be expected that different strains of SARS-CoV-2 may have different tendencies to induce it. To this end, it is reasonable that if microclots are important to LC their prevalence should also vary with the severity or frequency of LC induced by different strains of SARS-CoV-2. In a sense this would provide a very important kind of ‘control’, since the only thing varying as the stimulus is the strain of SARS-CoV-2.

### Other causes of amyloid microclots

While we here focus on SARS-CoV-2, we note that all kinds of molecules have been shown to affect the extent of fibrinaloid clot formation, including iron, other amyloids, bacterial cell wall components, etc., and that we have observed them in a variety of chronic, inflammatory diseases including Alzheimer's [37,50,60–62], Parkinson's disease [37,48], Type 2 diabetes [37,38,62,63,213,214] (where the amyloid protein amylin [215–217] is of course a well-known player) and rheumatoid arthritis [65,67]. Although not yet tested directly, we consider it likely that this will also be true for infectious diseases known to be causing similar post-infection syndromes, such as Dengue [218–220], Ebola [221–223], Lyme [224], Zika and others where viruses persist and can cause microangiopathies [139] that we suspect are also amyloid in character. There is also likely to be a role for molecules raised in pre-existing inflammatory diseases, as well as substances produced by dyregulation microbiomes [225], and Leiden factor V [226,227].

## Similarities to ME/CFS

As well as the post-infection diseases referred to above, the emergence of long COVID has brought to the fore its similarities to other even more widely established syndromes such as myalgic encephalomyelitis/chronic fatigue syndrome (ME/CFS) [1,228], and full details are available in these recent reviews [1,228]. Berg and colleagues have also highlighted the role of coagulopathies in ME/CFS [229]. We note, too, that other ‘anomalous’ diseases bearing symptoms that overlap with those of ME/CFS include Gulf war syndrome [230], where again we would hazard that the analysis of fibrinaloid microclot formation would have a positive outcome. Thus, while we consider it likely that the phenomena we describe will also broadly be true for ME/CFS, we focus here on PASC/Long COVID.

## Endotheliopathy

An important component of severe acute COVID-19 disease accompanying pathological clotting, is virus-induced endotheliopathy resulting in systemic endotheliitis [111,231–235]. Endotheliitis is central to initiating a state of failing normal clotting physiology and it is also known to be significantly linked to coagulopathies, as it activates microthrombotic pathways and initiates endotheliopathy-associated intravascular microthrombi [236].

## A role for iron dysregulation

A consequence of cell death, such as occurs in endotheliopathy, is the release in COVID patients of the normally intracellular iron storage protein ferritin [237,238], which can then release free iron [239]. Iron dysregulation is an accompaniment to a huge number of chronic, inflammatory diseases [200,240,241], and iron dysregulation is a significant accompaniment in COVID [238,242–245]. Indeed, there is evidence that SARS-CoV-2 can release iron from haemoglobin directly [246]. The involvement of iron dysregulation would be consistent with the potentially protective effects of chelating it, e.g. with lactoferrin [247–250] or other chelators [251–254].

## Role of platelets

**Figure 9.**
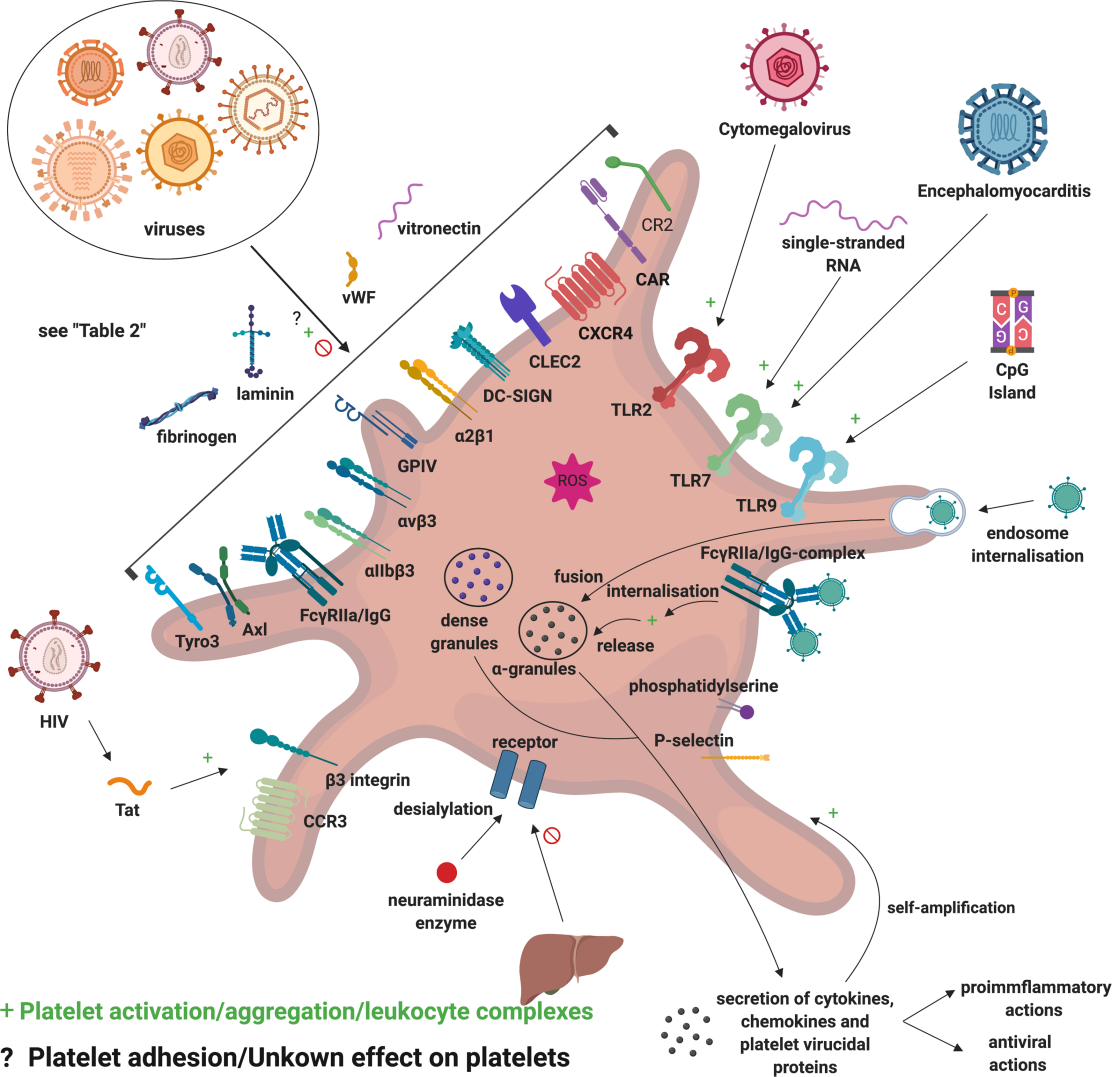
Platelet interactions with viruses. Figure adapted from [259]. Various platelet receptors can mediate binding to viral particles, where pattern recognition receptors recognize viral signals, viral products and also modulate platelet function. Platelets mediate viral attack by secreting virucidal proteins and by engulfing viral particles, as well as by interacting with immune cells and enhancing the immune response. Virus-platelet aggregates and platelets with a viral load are targeted by leukocytes, and platelets are ultimately cleared from the circulation (Figure created with https://biorender.com/).

The important role that platelets play in acute COVID-19 (Figure 9), has been discussed in the context of disease severity, the development of endotheliopathy and as general drivers of coagulation pathology [232,233,255–257]. Platelets interact with circulating inflammatory molecules, the (damaged) endothelium itself, and also with immune cells, resulting in platelet/cellular and platelet/molecule complexes [258,259]. Platelet complexes are mediated by membrane-membrane interactions via receptor binding. Two of the molecules central in this discussion are fibrinogen and VWF, and platelets form significant complexes with them both [260]. Ultimately, platelet–cell and platelet–coagulation molecule protein complexes form part of platelet activation mechanisms and vascular remodeling and these complexes drive granule secretion, surface glycoprotein expression, and molecular activation platelet hyperactivation pathways [258,259].

## Consequences for treatment protocols

Given that there is a definite background removal rate of fibrinaloid microclots, albeit slower than that of normal non-amyloid clots, it is then mostly necessary to ensure that they do not form further. Consequently, we do not see a role for ‘clot-busters’ as have occasionally been used when acute COVID-19 is accompanied by Acute Respiratory Distress Syndrome [261]. However, addressing only one of the pathologies (either clot pathologies or platelet hyperactivation) will always tend to fail, as was also noted in recent clinical trials where just one of the two therapies was trialed in Acute COVID-19. Lawler and co-workers [262] investigated the use of therapeutic anticoagulation with heparin in noncritically ill patients with COVID-19. (We note too that heparin, like lactoferrin [250], may have antiviral properties [263–266].) Lopes and coworkers [267] also investigated the use of therapeutic versus prophylactic anticoagulation for patients admitted to hospital with COVID-19. Earlier, Viecca and colleagues reported [268] on a single-center, investigator-initiated, proof-of-concept, case control, phase IIb study. The study [268] explored the effects of anti-platelet therapy on arterial oxygenation and on clinical outcomes in patients with severe COVID-19 with hypercoagulability. Outcomes were not significantly positive in any of the trials mentioned.

However, it must be noted that the trials were done on acute COVID-19 patients. This has led us to suggest a multipronged approach and a regime of triple anticoagulation treatment [231], where Long COVID patients might be treated by one month of dual antiplatelet therapy (DAPT) (Clopidogrel 75 mg/Aspirin 75 mg) once a day, as well as a direct oral anticoagulant (DOAC) (Apixiban) 5 mg twice a day, together with a proton pump inhibitor (PPI) (e.g. pantoprazole 40 mg/day for gastric protection). Such a treatment regime showed promise under condition of clinical practice (there were no treatment-free ‘controls’) [231]. However, especially because of the potential for hypocoagulation (bleeding), it must only be followed under strict and qualified medical guidance in which we recommend the regular assessment of coagulation status before and during treatment. It (as do any other methods [269]) now needs to be studied in a proper randomized controlled trial.

We do note that the use of triple therapy is not new in clinical practice. Its uses predate COVID-19, where it is successfully prescribed in patients where thromboses (which may or may not involve fibrinaloid clots) are particularly worrisome and in various coronary diseases accompanied by atrial fibrillation. Here strong anticoagulant treatments tailored to the needs of the individual patient are recommended [270], commonly involving dual or triple treatments with various anticoagulant agents. The present authors have focused on the ‘triple treatment’ for anticoagulation that involves an oral anticoagulant plus two drugs designed to decrease platelet activation (usually the P2Y_12_ inhibitor clopidogrel, plus low-dose aspirin). (In addition, a gastric proton pump inhibitor is also given to reduce the likelihood of gastric bleeding.)

## What is needed next?

Many pieces of research-level evidence (especially [85,101,106,108,109,116,231,271]) suggest strongly that fibrin amyloid microclots, driven by the presence of the SARS-CoV-2 spike protein, are an inevitable accompaniment to (and a likely cause of) Long COVID. A biologically coherent explanation [272,273] can link such observations with the other observable symptoms of Long COVID, and thus serve to satisfy the logic normally required [179,274,275] to provide a causative explanation.

However, we are still potentially far from translating this kind of understanding into both diagnostics and therapeutics. Some of the elements that are needed both to make the evidence even more robust and to pass regulatory approval more widely include the following:

- Our fibrin amyloid diagnostic methods, presently semi-quantitative, must be subject to further optimization, made robust and quantitative, and reproduced more widely
- Work is required to provide a simple, cheap and widely available point-of-care instrument to effect the necessary measurements of the number, size and nature of fibrinaloid micrclots
- Longitudinal studies, and those relating fibrinaloid presence to the severity of Long COVID, will help recognise the role of different fibrinaloids in causing different symptoms; these may vary predictively between SARS-CoV-2 strains
- There would be value in assessing other blood parameters simultaneously (e.g. fibrinogen, d-dimer, von Willebrand Factor) plus suitable inflammatory cytokines
- Armed with the above, we should reasonably expect clinicians to be able to obtain ethics for a suitable designed Randomized Controlled Trial of anticoagulant and platelet inhibitor regimes, coupled to the assessment, using viscoelastic assays such as Thromboelastography (TEG or ROTEM or Sonoclot), before and during such treatments of coagulation potential so as to guard against any hypocoagulation and the dangers of bleeding.
- Such individuals should be followed for a period after the end of treatment to assess the degree of permanence

Of course it is easy to design detailed and expensive trials, accompanied by a great many other measurements of covariates, but the above sets out what we would consider as the minimum necessary to strengthen the claims that the analysis and removal of fibrin amyloid microclots should have major utility in providing substantial benefits to those with Long COVID, and likely in related conditions as well.

## Conclusions

**Figure 10.**
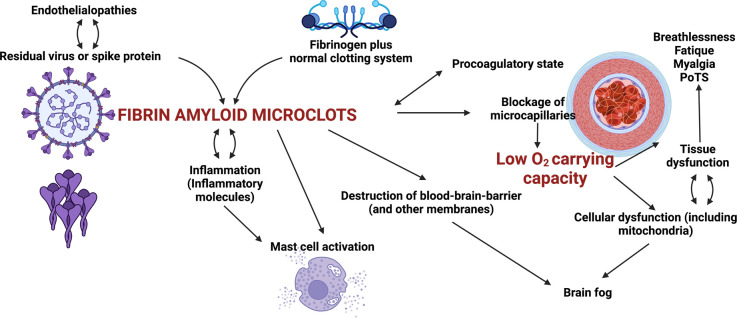
Some of the sequelae of fibrinaloid microclot formation in the symptomology of Long COVID. Many others, such as a role for auto-antibodies, are not shown.

Here we have argued, and focus on the fact, that Long COVID is characterized by the presence of persistent fibrin amyloid microclots that might block capillaries and inhibit the transport of O_2_ to tissues, entrapping numerous inflammatory molecules, including those that prevent clot breakdown (as we have indeed recently shown) (Figure 10).

In addition to microclot formation, significant platelet dysfunction and a systemic endotheliitis drive systemic cellular hypoxia. These pathologies can explain most, if not all, of the lingering symptoms to which individuals with long COVID refer. We have noted that amyloid microclots, platelet hyperactivation and endothelial dysfunction, might form a suitable set of foci for the clinical treatment of the symptoms of long COVID [231]. Therefore, if fibrinaloid microclots are largely responsible for the symptoms of Long COVID, their removal is to be seen as paramount for relieving these symptoms and allowing the body to repair itself.

## Open Access Statement

Open access for this article was enabled by the participation of University of Liverpool in an all-inclusive Read & Publish pilot with Portland Press and the Biochemical Society under a transformative agreement with JISC.

## Abbreviations

DAPT: dual antiplatelet therapy
DIC: disseminated intravascular coagulopathy
INR: International normalized ratio
LPS: lipopolysaccharide
PASC: Post-acute sequelae of COVID
PPP: platelet-poor plasma
TEG: Thromboelastography
